# Supplementary material for: Genetic Mapping of Flavonoid Grain Pigments in Durum Wheat
Source: Plants (Basel). 2023 Apr 17;12(8):1674. doi: 10.3390/plants12081674 (PMC10142998; doi:10.3390/plants12081674)
Supplement: Supplementary file 1 [file plants-12-01674-s001.zip › plants-2312778-supplementary Figures S1-S4.pdf]

Supplemental Figures

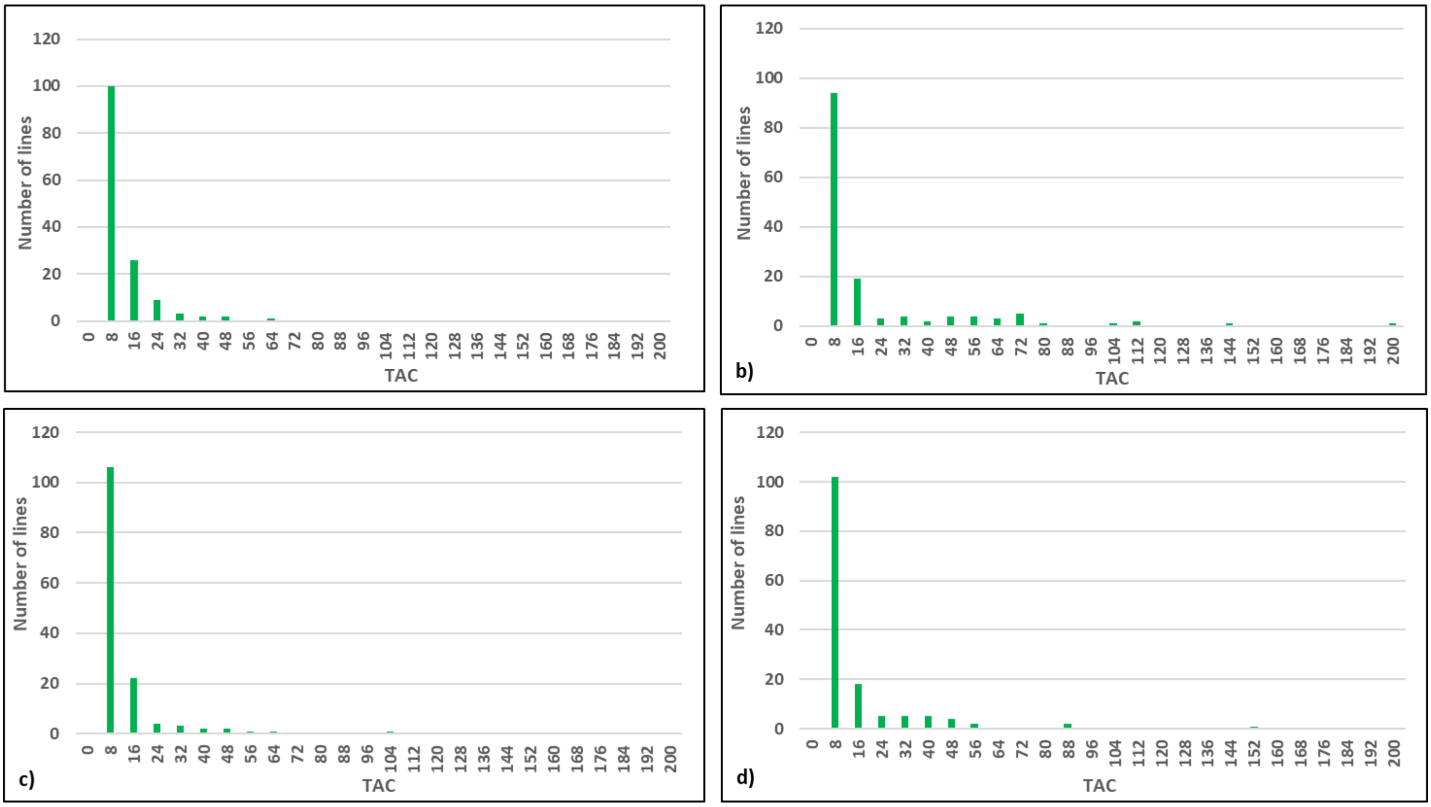

Figure S1. Frequency distributions of total anthocyanin compounds (TAC) in four field trials carried out at Valenzano 2020 (a), Valenzano 2021 (b), Bari 2021 (c), Valenzano 2022 (d).

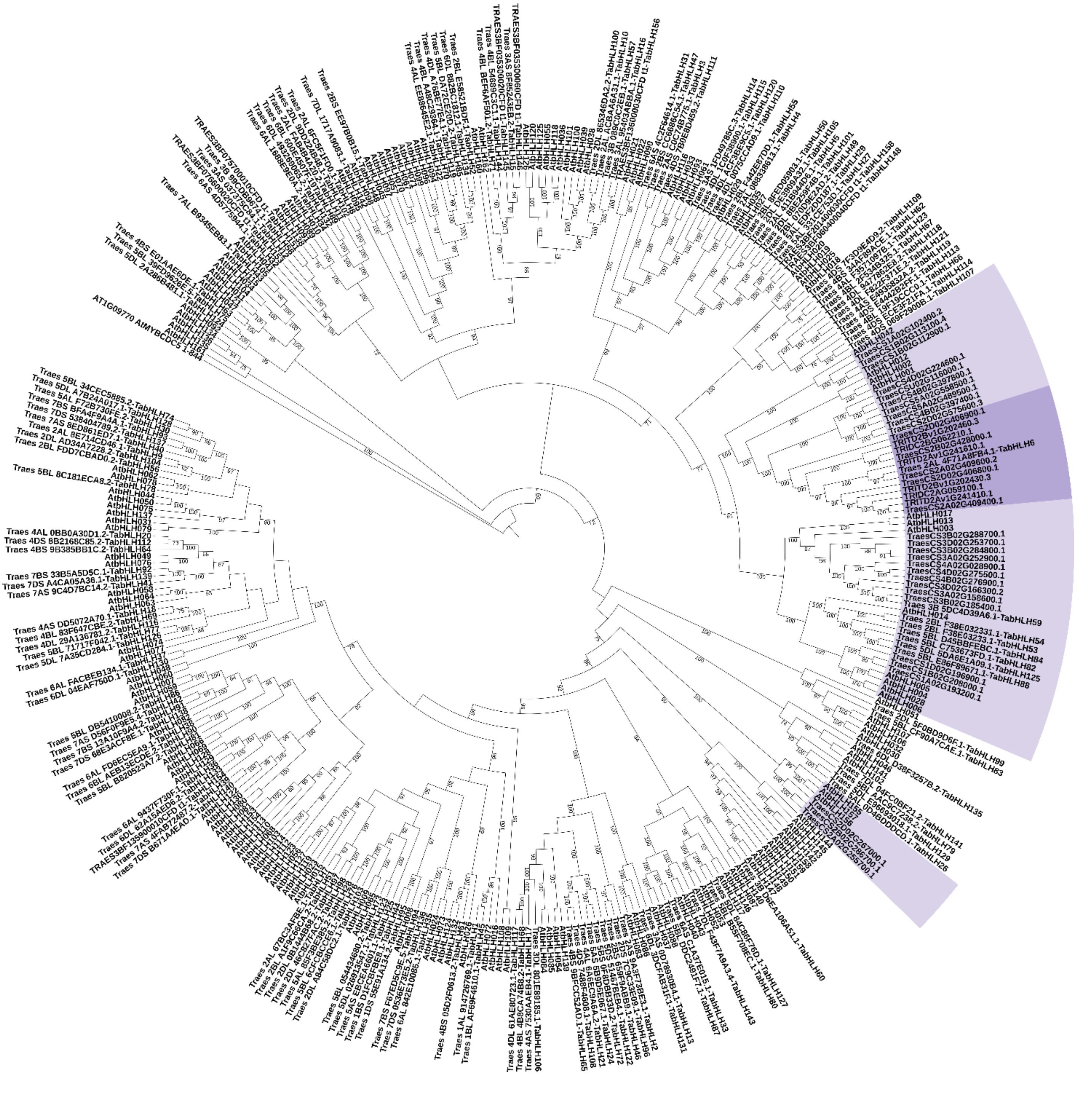

Figure S2. Phylogenetic analysis of bHLH proteins from wheat and *Arabidopsis thaliana*. Maximum Likelihood tree including the putative durum TFs (TRITD) identified in this study, their wild emmer orthologs (TRIDC), bread wheat TabHLH and subclass TaMYC proteins (TraesCS), and the *Arabidopsis thaliana* AtbHLH TFs. Purple: putative MYC proteins; dark purple: group with the MYC candidate genes here identified. The tree is rooted with AtCDC5 protein. Numbers on the branch nodes indicate bootstrap values (only bootstraps > 60 are shown).

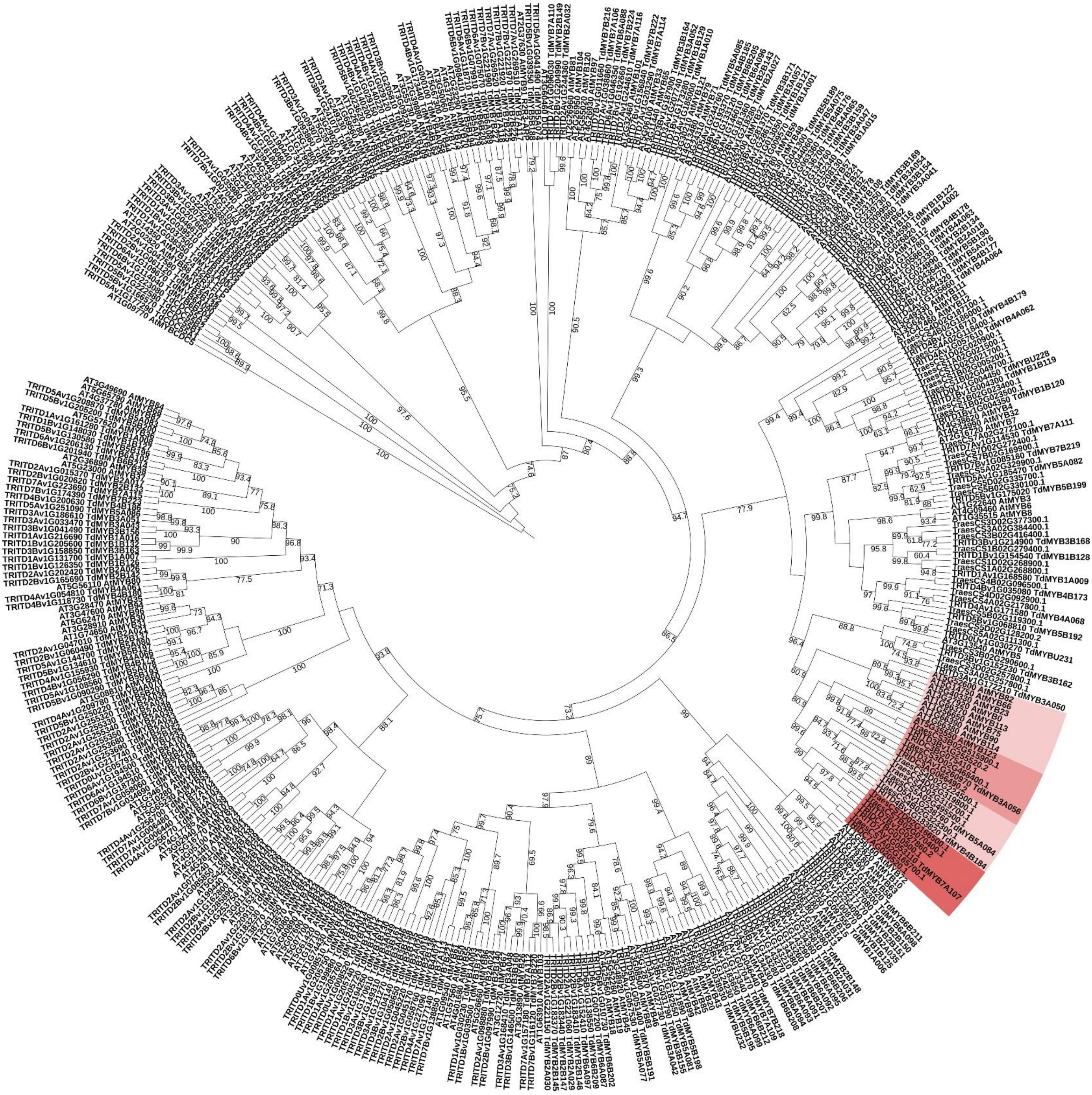

Figure S3. Phylogenetic analysis of R2R3-MYB proteins from wheat and *Arabidopsis thaliana*. Maximum Likelihood tree including durum wheat (TRITD), their wild emmer orthologs (TRIDC), bread wheat (TraesCS) proteins putatively involved in the regulation of phenylpropanoid biosynthesis, and *A. thaliana* AtMYBs. Brown: group with the putative MYB genes identified in this study. The tree is rooted with AtCDC5 protein. Numbers on the branch nodes indicate bootstrap values (only bootstraps > 60 are shown).

```
Norin61                  GGCCAAGCCGCCGCCGCCGCCGCCGCAGGCCGGCGGCGAGGCGCACGCCCTGCCCGAGGGCACGA
Chinese Spring           GGCCAAGCCGCCG-------------------GCGGCGAGGCGCACGCCCTGCCCGAGGGCACGA
Svevo                    GGCCAAGCCGCCG-------------------GCGGCGAGGCGCACGCCCTGCCCGAGGGCACGA
Zavitan                  GGCCAAGCCGCCG---------CCGCAGGCCGGCGGCGAGGCGCACGCCCTGCCCGAGGGCACGA
                         *************                   *********************************
```

Figure S4. Alignment of *Tamyb10-B1* gene sequences from the red-grain bread wheat cv. Norin 61 [43], bread wheat Chinese Spring, wild emmer Zavitan, and durum wheat Svevo.
